# Report of D. W. Hammond, M.D., Chairman of the Committee on Surgery for the Eighth Congressional District

**Published:** 1873-06

**Authors:** 


### REPORT OF D. W. HAMMOND, M.D., CHAIRMAN OF THE COMMITTEE ON SURGERY FOR THE EIGHTH CONGRESSIONAL DISTRICT.

*To the President and Members of the Georgia Medical Association:*

At the last meeting of the Association I had the honor to be appointed Chairman of the Committee on Surgery for the Eighth Congressional District. I know but little of the surgery of the district, with the exception of a few cases in the city of Macon and immediate vicinity. I have heard nothing from my colleagues, Dr. B. F. Rudicil, of Monroe county, and Dr. C. L. Strother, of the county of Pike.

My esteemed friends and confreres, Drs. C. B. Nottingham and G. E. Sussdorff, have each kindly furnished me with a case of surgery in their own practice: Dr. Nottingham—Ovariotomy; Dr. Sussdorff—Tracheotomy. An account of each of the above operations has been written out by themselves, which I take great pleasure in tendering to the Association.

In addition to these cases, I also beg leave to report a case which occurred in Vineville, a small village in the immediate vicinity of the city:

*Cutting Throat.—Operation Unsuccessful, and Performed by the Patient.*

Mrs. Bone, aged twenty-four years, phlegmatic temperament, low in stature, and the mother of three children, on the eighth day of December last, at 8 o'clock A.M., attempted to commit suicide by cutting her throat with a razor. With a determined effort and a sweeping stroke of the instrument she severed completely all the tissues of the neck to the cervical vertebræ. The wound extended from the sterno-cleido-mastoideus muscle of the right to the corresponding muscle of the left side. The external jugular veins on both sides were divided and the carotid arteries laid bare. The pulsations of these vessels could be distinctly felt by introducing the finger into the opening. The *trachea* and œsophagus were both cut across, above the *pomum adami*. The razor being directed upward, severed completely the integuments above the *glottis* and *epiglottis*, and passing over the *os hyoides*, just beneath the base of the tongue, detaching these important organs from the *larynx* and *pharynx*. The *pharynx*, at its inferior portion, was cut through just at its junction with the œsophagus, and extended through all the intervening tissues to the cervical spine, as before stated. The organism thus transfixed, retracted, or rather dropped down to the top of the sternum, leaving a large and frightful gash from three to four inches in extent.

As the *os hyoides* and *rima* were separated from their site, all the muscles that arise from the different cartilages of the trachea, and which are inserted into the hyoid bone, were cut through; consequently their several offices were suspended, which was well illustrated by the pathological effects upon the physiological action. These eight little muscles, arising from as many different points, are all inserted into this bone, and when brought into action move it in every conceivable direction; they elevate and dilate the *pharynx* to grasp the food, then contract to propel it into the gullet. From such a lesion, deglutition, as well as the voice, was for the time interrupted. Under such circumstances she was sooner or later to realize the fearful prospect of starvation. The vocal cords being silent, she could not utter a word, or an intelligent sound by which to make her wants known; could not eject the sputa from her mouth and throat. In such a dilemma, she was in constant dread of suffocation.

Thus circumstanced, her only means of sustenance were to be attained through other channels, and her wishes to be perceived by *signs* and *writings*. After inflicting the wound, she passed out of the room and across a hall into another apartment, where she was found by her husband lying apparently in a lifeless condition. At which place I presume she swooned, from the large quantity of blood found upon the floor, and upon her person. She was bathed with a cold, clammy sweat; pulse flickering and almost imperceptible at the wrist; surface blanched; respiration hurried and labored, with bloody froth issuing from her mouth and from the wound.

As usual in such cases, messengers were dispatched in quest of doctors. In a short time some four or five were in attendance. The patient was supplied with dry clothing—the wound cleansed—and then placed in a warm bed. The prognosis was so unfavorable for recovery that the physicians merely directed her to be kept quiet, and to use all the usual applications requisite to restore the normal temperature of the body, and to establish reaction.

Saw her again at 4 P.M., and found that reaction had been partially established. Pulse quite feeble, but firm, and eighty per minute. By next morning she had so far recovered as to recognize her friends, and to fully realize her perilous situation, and made her wants known by "signs and writing," as before stated.

Her mouth and tongue were parched and dry. To assuage her thirst, cold water was frequently taken into her mouth, which readily passed out through the wound beneath the chin. On the third day she was harrassed with insatiable thirst and hunger, and begged for something to be done to ameliorate her sufferings.

Drs. Holt, Nottingham, Fitzgerald, Mettauer, and myself, made every effort to introduce a tube into the œsophagus, to supply her with nourishment. But in all our most diligent and persevering efforts we were completely foiled.

On consultation it was determined to supply her with nourishment by nutritious enemata, and leave the case to nature. She was sustained in this way for about eighteen days, when it was discovered, to the great satisfaction of herself and friends, that a part of the fluids taken into the mouth did pass into the stomach. Finding that she could swallow fluids, her efforts became more persevering to supply the stomach with food. In the course of eight or ten days from this time her deglutition was so much improved as to admit solids to enter the œsophagus. This could not, however, be accomplished without assuming the recumbent posture. The enemata were now dispensed with.

She has gradually improved in health and strength, and in swallowing. The ingesta now readily pass into the stomach in the erect position.

After the lesion of the vocal apparatus, complete aphonia was the result. The voice, as the opening contracted, gradually but imperfectly commenced returning. I discovered that she uttered monosyllables beginning with a vowel with more clearness than those commencing with a consonant. Consonants are always more difficult to enunciate than vowels. I saw her last week, and although her articulation is still imperfect, yet she can be understood sufficiently to comprehend what she says. Her voice is gradually improving. The interesting but difficult problem now presents itself: What means were instituted by nature to restore the voice? The factors engaged in the sounds uttered by the voice were entirely destroyed. The glosso pharyngeal, and doubtless both of the recurrent nerves were divided—in fact, all the nerves distributed to the vocal apparatus.

The course the symptoms took in this case proves one fact beyond all doubt—that nerves divided do reunite.

Sir Everard Home and others have shown that when a nerve cord has been cut into, and extremities separated, it regains in a short time the power of transmitting the normal nervous influence.

In conclusion, I am of the opinion that wounds of this kind should be left to nature. Surgical interference is useless, and would be injurious rather than alleviative. Of course, if an artery is severed and bleeding, it should be ligated. In addition to this, the head should be kept well forward, with the chin pointing to the sternum. Wound to be properly dressed, kept clean, etc.

In speaking of this subject, Sir Astley Cooper remarks "That the introduction of tubes into *pharynx* and *œsophagus* are worse than unnecessary, for they are highly injurious, by the cough which they occasion irritating the wound; and if adhesion or granulation has taken place close to the wound, such tubes tear it open again, and destroy the process of restoration." The correctness of this opinion is fully borne out by the result of the above case.

The wound has entirely closed, except a small fistulous opening above the thyroid cartilage. The air passes through this opening, and occasionally a few drops of water.

I presume the fistula will continue patent until the appropriate operation is resorted to for its occlusion. The operation usually recommended is simply to pare the edges of the opening, and then bring the lips together by two points of suture. Astly Cooper says "that, in a wound of this kind which remained fistulous, he raised a piece of skin from the surface of the neck, above the fistulo, and turned it over it, having previously pared the edges, and it united well."

### *Rapid and Immense Cystic Collection—Ovariotomy—Death. By* C. B. Nottingham, M.D., *Macon, Ga.*

Mrs. D., of Houston county, came to Macon November 7th, last year, with the view of having me operate for ovarian disease. She was thirty-four years of age, and the mother of four children—the youngest being five years old.

I learned that she had enjoyed fair health until about two and a half years before her visit to me, when her menses became disordered, and that soon thereafter a lump appeared low down in the left iliac region. The tumor was painful, and increased slowly but steadily in size. The uterine hemorrhage soon became almost continuous. Her general health began to give way, and ere long it was found necessary to command the services of a physician. An ovarian tumor was diagnosed by her adviser and a protracted course of medical treatment was instituted—sometimes attended with hope that her condition was being bettered, but ultimately with the conviction that surgical means must be invoked. During the last few months she had grown rapidly in size—had attained such burthensome proportions that, on her arrival in Macon, her abdomen projected to her knees, and gave a circumference at the umbilicus of sixty-one inches, and a length, from ensiform cartilage to pubis, of forty-two inches. Dullness on percussion, with a wave-like motion of fluid under palpation, obtained generally over this immensely distended abdomen. High up in the epigastrium only was there resonance. Emaciated and feeble, her small thready pulse beat 120 per minute. Oppressed in her respiration when sitting, she had not been able to lie down for some weeks.

The accumulation of fluid was such as prevented the tumor being felt, and I determined, after consultation with my friends, Drs. Hammond, Fitzgerald, and Burgess, simply to tap for the present. This I did on the 10th, and drew off by measure 13½ gallons of straw-colored albuminous fluid—weighing 9 pounds to the gallon. The abdominal walls, at the point of puncture, were but little thicker than hardware paper, and would probably have yielded in rent very soon, had not the great distention been relieved. For two days she suffered a good deal of pain, for which warm stupes were used, and paregoric administered—an idiosyncracy, as she informed me, rendering the stronger preparations of opium unsafe. On the sixth day, she being quite comfortable, aided by the gentlemen above named, I made a careful examination, and discovered an uneven tumor, filling the left side of abdomen and pelvis—apparently about five by nine inches in diameter. Four days afterward she left for home with such medicine as her condition required—advised that she should be tapped by her family physician as soon as the accumulation of fluid should produce distress. This soon occurred, and on the 15th of December 8¼ gallons were evacuated. Reaccumulation taking place again very rapidly, she returned to Macon, and on the 16th January I removed 8¼ gallons more—the same in color and quality as in the first instance—making 30 gallons in two months and six days, the weight of which was 270 pounds.

Only a few days elapsed before there was evidence of recurring distension, and it being quite certain that she could not long survive under such a fearful drain from her system, and that nothing short of the radical operation she desired on first coming to Macon offered the slightest ground of hope of success in dealing with her malady, I felt it my duty, after consulting with the gentlemen who had been kind enough to see her with me, (although the case was most unpromising for the knife,) to give her the slight chance of cure that extirpation might afford. Having endeavored during a few days of preparation to induce a tolerance of opium, and having taken the usual preliminary measures in regard to the bowels, I proceeded on Saturday, the 1st day of February, to effect the operation—present and kindly assisting, Drs. Hammond, Fitzgerald, Burgess, Green, Magruder, Wright, and Sussdorff. An effort was made to induce anæsthesia with ether, but it having failed to impress her in a reasonable time, and distressing nausea being induced by it, chloroform was substituted. As soon as insensibility was effected, an incision 3½ inches long was made. Examination through this wound revealed adhesions between what appeared to be a large cyst and the peritonæum, about the points of the several tappings. It proved to be a cyst, and so thin were its walls that, under the most gentle effort to break off these adhesions with the fingers, the cyst gave way and the fluid came pouring out. She being turned on her side, it was allowed to flow into a tub at the edge of the couch. A tumor of multilocular cysts was then discovered, and the wound was at once enlarged to eight inches. Four or five of the larger sacs were evacuated by incision as they presented, when the remaining mass was lifted out. The only adhesions found beside that already mentioned, were to the omentum and to the uterus. These were easily broken up with the hand. The pedicle was remarkably short, and, although embracing the entire broad ligament, was so small that it was thought proper to throw around it a single ligature composed of four strands of saddler's silk. This was unfortunate, as from some cause it slipped off soon after severance of the pedicle was effected. Seizing the stump with my fingers and thumb, it was immediately transfixed with a double ligature, and either half tied separately. With a view to drainage the ends were left long, and brought out at the lower angle of the wound. The loss of blood hardly amounted to a pint, and was carefully sponged from the pelvis, with some cystic fluid that had gravitated to that point. Before closing the wound, I deemed it expedient, as a measure of prospective drainage, to pass a tube through the *cul-de-sac* of Douglas. This having been done, and the right ovary having been examined and found healthy, the wound was drawn together with sutures of silk, entered from within, including the edge of the peritonæum, and being brought out an inch from the line of incision. The usual dressing was applied, and she, having aroused from the chloroform, was given one grain of gum opium, (that preparation having been found to agree with her best when the effort was made to produce tolerance,) and put to bed at 1½ o'clock—pulse 90, respiration 16 per minute. Ordered lumps of ice occasionally, and one ounce of beef-tea or iced milk, at her pleasure.

The anæsthetic was borne quite badly, it being necessary to suspend the operation several times because of the violent retchings and vomiting.

The solid tumor weighed 6½ pounds, and the fluid caught in the tub measured 7¼ gallons, weighing 9 pounds to the gallon. Tumor and fluid weighed 71¾ pounds, which, with the fluid removed at the three tappings, amounted to 343 pounds.

She reacted pretty well by 4 o'clock, and quite well by 7 o'clock P.M. Slept well during the night, and promised well during the next day and night, but Monday morning, at 5 o'clock, she had a chill that was followed by fever. Symptoms of well-pronounced peritonitis were speedily developed, and she steadily grew worse until 4½ o'clock Wednesday morning—88 hours after the operation—when she expired. Up to the time of the inauguration of peritoneal inflammation, she was simply nourished with beef-tea and milk and crackers, had her bladder emptied every twelve hours, and got half grain of opium every three hours. On the occurrence of peritonitis, the drainage tube was gently moved in various directions, with the effect, however, of discharging only a little offensive gas, when warm water was freely thrown into the abdomen and pelvis. This was followed by the escape of some fœtid, pink-colored fluid. The quantity was small, and the injection of warm water was continued until it returned clean, when salt-water was substituted, and continued from time to time until her condition became hopeless. The opium was given more freely, whisky punch was liberally administered, and warm stupes applied, but all in vain.

*Tracheotomy. By* G. E. Sussdorff, M.D., *Macon, Ga.*

On the 25th of last August, I was invited by Drs. D. W. Hammond and Mason, of this city, to see a child, Thomas Cooper, aged 4 years, who was suffering from the presence of a foreign body in the trachea, which had gained entrance some eighteen days previously.

Before the accident, the health of the child was good, but at the time of my visit he was very weak and emaciated; had considerable fever, and I learned from Dr. Mason he had had several rigors. Pulse 130 per minute, and small. His appetite was bad, and but little nourishment could be taken. His breathing was short and hurried, interrupted frequently by violent fits of coughing, threatening asphyxia.

The ear applied to the course of the trachea *discovered* the foreign body floating up and down, sometimes striking violently against the larynx. The lungs were healthy, but there was considerable bronchitis.

Every effort to discharge the foreign body having proven unsuccessful, the operation of tracheotomy was determined upon as the only chance for his life.

The next day Drs. Hammond, Burgess, and Mason met me again, and we proceeded to the operation, by chloroforming the patient. All the various steps laid down by Erichsen and Gross for the operation of tracheotomy were carefully observed.

The incision through the integuments extended downward, about an inch and a half from the cricoid cartilage, in the mesial line. Careful dissection followed, and all went well until the trachea came into view, when one of the veins of the thyroid-plexus was cut, and hemorrhage became rapid. The patient coming from under the influence of the chloroform began to cough, and with each expiratory effort the blood was forced out in jets.

An attempt was made to arrest the flow, but proving unsatisfactory, it was deemed best to proceed with the operation, and

I at once made an incision from the second ring to the cricoid cartilage. The patient having been quickly turned upon his stomach, it became evident in a few seconds that the opening was too small, and that he was suffocating.

He was again quickly turned upon his back, the trachea steadied with the finger in the wound, and the incision carried through the cricoid cartilage.

Instantaneously with the incision a *grain of corn*, much swollen, was thrown out through the wound, followed by a large quantity of thick, tenacious mucus; a good deal also came away through the mouth.

The air now passed through the opening *freely*, and a few moments later the hemorrhage ceased. The patient was kept upon his stomach for a while, and ice applied to the edges of the wound to check any internal bleeding from the membranes that might be going on. The time from the second incision to the establishment of free respiration was about half a minute.

The patient was allowed to breathe through the wound some little time. A little toddy was occasionally given, and when reaction was pretty well established, the wound was closed by three sutures, and the dressing completed with adhesive plaster, a cotton compress and bandage.

One hour after the operation the patient, in a voice almost natural, asked for nourishment. He improved at once, and on the 10th of September the wound had healed finely throughout.

I have seen the child several times during the past year, and he appeared quite well; *voice natural*, and the line of incision scarcely perceptible.

I do not claim anything extraordinary about this case, or the operation, but I am convinced from the circumstances in this instance:

1st. That much of the danger in this operation is consequent upon the smallness of the opening made.

2d. That when hemorrhage occurs during the operation, from blood vessels external to the trachea, the cut through the windpipe should be large enough to admit of the free passage of air, and should not be delayed.

3d. That in all cases where time is of vital importance, if an incision were made at once, in the upper part of the trachea, directly through the *integuments and trachea*, and sufficiently extensive to admit the air freely, it would be as free from danger and give more satisfactory results than a more careful dissection of parts. Indeed, from the history of many cases of attempted suicide, by cutting the throat transversely, it would appear that the *transverse section* is the safest plan after all—such cases rarely proving fatal.

